# Dementia Ready: Comparing Approaches for Building Caregiver Readiness and Knowledge of Dementia

**DOI:** 10.1093/geroni/igab046.3476

**Published:** 2021-12-17

**Authors:** Michael Lepore, Kate Keefe, Erica DeFrancesco, Julie Robison, Alis Ohlheiser, Christine Bailey, Kathy Kellett

**Affiliations:** 1 LiveWell Alliance, Southington, Connecticut, United States; 2 LiveWell, Plantsville, Connecticut, United States; 3 University of Connecticut, University of Connecticut, Connecticut, United States; 4 UConn Health, Farmington, Connecticut, United States; 5 University of Connecticut Health Center, Farmington, Connecticut, United States

## Abstract

Despite the rising prevalence of dementia and the high cost and complexity of care for people with dementia, most dementia care is provided at home by informal caregivers who are not clinically trained. Building caregiver readiness and knowledge of dementia is key to supporting quality care and desirable health outcomes, such as preventing falls and reducing nursing home admissions. We sought to determine and compare the impact of two interventions—Resilient Living with Dementia (RLWD) and Care of Persons with Dementia in their Environments (COPE)—and of their combined delivery (both RLWD and COPE) on increasing caregiver readiness and knowledge of dementia. Between January 2019 and March 2021, 77 caregivers of people with dementia in Connecticut participated in RLWD and/or COPE and completed the Alzheimer’s Disease Knowledge Scale (ADKS) and the Preparedness for Caregiving Scale (PCGS) at baseline and at four-month and ten-month follow-ups. Analyses were conducted to compare outcomes by intervention(s). From baseline to four months and to ten months, we observed statistically significant (p < .05) improvement on the ADKS among participants in RLWD, and on the PCGS among participants in COPE and among participants in RLWD. The most substantial impact on PCGS was observed among participants in both COPE and RLWD. No improvement in the ADKS was observed among participants in only COPE, but ADKS improvement was observed at four months among participants in COPE and RLWD. Findings suggest that the benefits of COPE and RLWD for building dementia caregiver readiness are complementary and mutually reinforcing.

